# Teen dating violence: predictive role of sexism and the mediating role of empathy and assertiveness based on gender

**DOI:** 10.3389/fpsyg.2024.1393085

**Published:** 2024-06-19

**Authors:** Víctor José Villanueva-Blasco, Begoña Iranzo, Joaquín Mateu-Mollá, Laura Carrascosa, Sandra Gómez-Martínez, Marta Corral-Martínez, Mª Teresa Mitjans, Mª Jesús Hernández-Jiménez

**Affiliations:** ^1^Faculty of Health Sciences, Valencian International University, Valencia, Spain; ^2^Research Group in Health and Psycho-Social Adjustment (GI-SAPS), Valencian International University, Valencia, Spain; ^3^Faculty of Social and Legal Sciences, Valencian International University, Valencia, Spain; ^4^HUCASAN – Humanizing Health, Quality and Healthcare Management Research Group, Valencian International University, Valencia, Spain; ^5^Research Group in Health, Violence and Trauma (GI-SAVITRA), Valencian International University, Valencia, Spain

**Keywords:** teen dating violence, sexism, empathy, assertiveness, adolescents

## Abstract

**Background:**

Despite efforts to prevent dating violence among adolescents, it remains a major problem with multiple negative consequences. Sexist beliefs, empathy, and assertiveness influence teen dating violence (TDV) with potential gender differences.

**Objectives:**

(1) Determine gender disparities in TDV perpetration and victimization, including relational, verbal-emotional, and physical aspects, as well as roles; (2) Analyze gender variations in sexism, empathy, assertiveness, and their relationship with TDV; (3) Establish a predictive model of sexism in TDV with empathy and assertiveness as mediators for both genders.

**Participants and setting:**

A sample of 862 secondary school students (50.2% females, 49.8% males; mean age: 14.1 years) from diverse regions in Spain participated.

**Methods:**

TDV was measured using the Conflict in Adolescent Dating Relationships Inventory (CADRI) in a cross-sectional study. Sexism, empathy, and assertiveness were assessed using the Ambivalent Sexism Inventory (ASI), Interpersonal Reactivity Index (IRI), and Assertiveness Inventory for Students Questionnaire (AISQ), respectively.

**Results:**

Females exhibited higher TDV perpetration, specifically verbal-emotional TDV. Males showed more relational TDV and hostile sexism, while no benevolent sexism differences were observed. Mediation models demonstrated sexism, assertiveness, and empathy as individual predictors of TDV, with varying mediation effects. Personal distress partially mediates the link between sexism and TDV perpetration or victimization in males, while practical personal ability mediates between sexism and TDV perpetration in females.

**Conclusion:**

Sexism predicts both perpetration and victimization in TDV, linked to empathy and assertiveness. Notably, specific dimensions of empathy and assertiveness mediate the connection between sexism and TDV, displaying gender-specific patterns. Preventive measures should consider personal distress in male perpetrators/victims and practical personal ability in female perpetrators.

## 1 Introduction

Currently, teen dating violence (TDV) and its continuation into adulthood are a social reality with serious consequences for the health of those who experience it (Johnson et al., 2015; Wincentak et al., 2017; Miller et al., 2018; Spencer et al., 2020). Authors like Basile et al. (2020) define TDV as a type of partner violence in romantic relationships during adolescence, including physical, psychological, and/or sexual abuse. This phenomenon is considered a current public health problem, so it is necessary to expand studies on the risk factors involved (Aizpitarte et al., 2017).

Early dating experiences can establish patterns of violence and unhealthy relationships (Muñoz-Rivas et al., 2022). Additionally, alcohol and drug use serve as risk factors for dating aggression (Muñoz-Rivas et al., 2010), alongside attitudes and beliefs that justify them. These relational patterns have short and long-term consequences for the comprehensive development and health of the involved adolescents (Wincentak et al., 2017; Miller et al., 2018). Furthermore, the prevalence of TDV has increased in recent years (Carrascosa et al., 2019; Cénat et al., 2022). Therefore, research on TDV is an area of interest and concern in the scientific community.

TDV refers to a wide range of harmful behaviors that occur within adolescent dating relationships. It can be psychological (e.g., emotional manipulation), physical (e.g., pushing, kicking), sexual (e.g., forced sexual activity), and relational (e.g., social control, gossip, social exclusion). Studies indicate that approximately one in two adolescents report experiencing at least one form of TDV in the past 12 months (Hébert et al., 2017). In Spain, Vives-Cases et al. (2021) reported that the overall prevalence of victimization due to teen dating violence (TDV) was significantly higher among girls than boys (34.1% vs. 26.7%). Additionally, the exposure to psychological violence was higher in girls (28.1%) compared to boys (21.0%). There were no statistically significant sex differences in the prevalence of physical and/or sexual violence, although the recorded proportion was higher among girls (16.0%) than boys (11.8%). According to data from the Centers for Disease Control and Prevention (CDC) in the US, TDV is a widespread issue among adolescents, with 8.2% of high school students reporting physical and sexual violence in their romantic relationships (Basile et al., 2020). TDV may represent a risk factor for experiencing violence in adulthood (Piolanti et al., 2023).

TDV is often bidirectional, with adolescents of both sexes perpetrating and being victimized by their partners simultaneously (Cava et al., 2020; Marcos et al., 2020; Théorêt et al., 2021). This bidirectional violence is predominantly psychological (Graña and Cuenca, 2014; Rubio-Garay et al., 2017), although other authors, such as Dosil et al. (2020), highlight that relational TDV is the most frequent.

Recent research indicates the presence of gender differences in TDV, although a consensus has not yet been reached. For instance, Dosil et al. (2020) reported a higher percentage of male perpetrators among Spanish adolescents, while female victimization rates were higher. Conversely, other authors, such as Valdivia-Salas et al. (2021), have found a higher percentage of female perpetration in occasional and frequent TDV or specifically in cases of verbal-emotional and physical violence (Esparza-Martínez, 2019). Additionally, studies suggest that psychological violence is more prevalent among females, while sexual violence is more common among males (Rubio-Garay et al., 2017; Espelage et al., 2022). Regarding victimization, Marcos et al. (2020) found no significant gender differences in any type of TDV victimization, except for physical violence, where males scored higher.

In addition to examining gender differences, TDV research has focused on identifying risk and protective factors. Consequently, this study investigates the role of sexist beliefs, empathy, and assertiveness as potential risk or protective factors for TDV.

### 1.1 Sexist beliefs

The presence of beliefs that justify violence in romantic relationships, such as gender stereotypes, negative attitudes toward women, and favorable attitudes toward violence, has been identified as risk factors for TDV (Rubio-Garay et al., 2015; Reidy et al., 2016; Hunt et al., 2022). Adolescents with maladaptive schemas may play a relevant role in victimization in dating relationships (Calvete et al., 2018). This is particularly observed among adolescents who hold accepting attitudes toward the use of violence in dating relationships (Jennings et al., 2017; Smith-Darden et al., 2017).

Sexist beliefs are prevalent among Spanish adolescents (Ferragut et al., 2017; Ramiro-Sánchez et al., 2018), encompassing both hostile and benevolent sexism. Both forms of sexism are associated with the justification and perpetration of various types of violence, including TDV (Carrascosa et al., 2018; Vives-Cases et al., 2021).

Regarding gender differences, Ayala et al. (2021) found that males scored higher than females on sexism, and females who had experienced TDV reported higher levels of hostile sexism than those who had never been in an intimate relationship. Conversely, Pazos-Gómez et al. (2014) found that high levels of sexism were associated with increased threats of sexual, relational, verbal-emotional, and physical violence in males, whereas in females, it was only associated with higher levels of relational violence. Rey-Anacona et al. (2017) also found significantly higher scores on hostile sexism among males than females. Furthermore, hostile sexism was linked to severe physical aggression perpetrated by females, while benevolent sexism was associated with mild aggression perpetrated by females.

In terms of the relationship between victimization and sexist beliefs, Marcos et al.’s (2020) study supports the association between victimization in relationships and sexist beliefs and romantic love myths, suggesting that these attitudes and myths can facilitate the initiation and perpetuation of TDV. This aspect has also been demonstrated in other studies (Fernández-Fuertes et al., 2018; Rey-Anacona and Martínez-Gómez, 2022).

### 1.2 Empathy

Among the psychological factors that can act as protective factors against TDV, high empathy is highlighted (Vagi et al., 2013; Davis et al., 2019). According to Wolfe et al. (2004), empathy can moderate the relationship between attitudes of violence justification, self-efficacy, and TDV. This finding is consistent with multiple studies that indicate empathic capacity as an inhibitory factor of aggression (Tur-Porcar et al., 2016; Deschamps et al., 2018; Song et al., 2018), although authors like Vachon et al. (2013) suggest that this relationship is moderated. On the other hand, Espelage et al. (2020) observed through a longitudinal study that male perpetrators of TDV had significantly higher levels of empathy compared to non-perpetrators, but over time, the perpetrators’ empathy decreased while non-perpetrators’ empathy increased.

The study of empathy in this field seems more appropriate from Davis's (1983) multidimensional model. This model differentiates between cognitive empathy (Fantasy and Perspective Taking), which is the ability to understand how others feel, and affective empathy (Empathic Concern and Personal Distress), which corresponds to the vicarious experience of feelings expressed by others. Several studies report that aggressive individuals exhibit lower cognitive empathy than non-aggressive individuals (Gantiva et al., 2018). Additionally, Berger et al. (2015) indicate that affective empathy inhibits aggression but not cognitive empathy.

Regarding specific dimensions of empathy, Van Heerebeek (2015) found that perspective-taking was negatively related to indirect aggression in both males and females. However, personal distress was positively related to indirect aggression in males and direct aggression in females. Authors such as Guzmán-González et al. (2014) observed that university students who engaged in psychological TDV had low levels of perspective-taking and empathic concern. Similarly, Valdivia-Salas et al. (2021) showed a positive relationship between high personal distress and TDV in physical and relational forms. Regarding the relationship between victimization and empathy, the results of Dodaj et al.’s (2020) study indicate that empathic concern and perspective-taking dimensions are significant predictors of victimization in sexual coercion and psychological aggression. In summary, not all dimensions of empathy have the same protective capacity against TDV perpetration.

### 1.3 Assertiveness

Assertiveness is recognized as a valuable interpersonal skill for expressing needs, fostering effective romantic relationships, obtaining support, and facilitating successful conflict resolution (Xia et al., 2018). The cognitive model of assertiveness proposed by Vagos and Pereira (2010) encompasses four interpersonal schemas or fundamental beliefs, reflecting individuals’ beliefs about their ability to express themselves and respond appropriately in different social contexts. These schemas include external emotional support, interpersonal management, practical personal ability, and affective personal ability. The first schema refers to a positive representation of others as suppliers of support, acceptance, and affection (e.g., “When I am sad, angry, or upset, I have someone to support me and help me feel better”). The second pertains to a representation of the self as possessing the abilities needed to manage daily life (e.g., “I am capable of performing tasks at work (or school) as well as most people”). The third relates to the ability to solve problems as part of interpersonal encounters (e.g., “When someone I like pulls away from me, I try to understand why and solve the situation”). The fourth conveys beliefs about the self being lovable (e.g., “I feel I am special to some people”).

Research on the role of assertiveness in TDV suggests that this skill can serve as a protective factor in escalating TDV conflicts. For example, Simpson-Rowe et al. (2012) found that assertive skills were associated with a reduced risk of TDV, particularly for young females, including sexual victimization and coercion. Similarly, Fortin et al. (2021) reported a negative association between assertiveness and TDV perpetration. However, Xia et al. (2018) did not find significant relationships between assertiveness and physical or psychological TDV. On the other hand, Valdivia-Salas et al. (2023), drawing on Vagos and Pereira’s (2010) cognitive model of assertiveness, indicated that practical personal ability was explicitly associated with high TDV perpetration or victimization among adolescents.

Gender differences in interpersonal skills during adolescence have been documented, with females generally exhibiting higher levels of general interpersonal skills than males (Salavera et al., 2019; Persich et al., 2020). Notably, Ayala et al. (2021) found that high assertiveness was associated with higher levels of benevolent sexism in both sexes and higher levels of hostile sexism in males.

Despite the extensive literature linking these variables to TDV, no theoretical model has been found that comprehensively considers all of them. Although the well-established link between sexist beliefs and TDV, limited attention has been given to understanding the role of empathy and assertiveness as mediators in this relationship. A model in this sense can contribute to developing preventive and treatment programs that cultivate interpersonal skills capable of moderating the impact of sexist beliefs on TDV perpetration and victimization. It is important to note that the relationships among these variables may be sex-dependent and can vary depending on the specific constructs examined.

Considering the previous literature that establishes the relationship of sexism, empathy, and assertiveness with TDV, are there gender differences regarding all these variables, and consequently, is it justified to develop a predictive model of TDV differentiated by gender? Assuming gender differences are found according to previous literature, does sexism predict both perpetration and victimization in TDV? Are empathy and assertiveness mediating variables between sexism and TDV in both directions, perpetration, and victimization? The main objective of the present study is to propose predictive models of sexism differentiated for perpetration and victimization of TDV, with empathy and assertiveness as mediating variables for both genders. To achieve this, several intermediate objectives are established aimed at confirming the conditions that support the formulation of such a model. Firstly, it examines whether significant gender differences exist in TDV perpetration and victimization, including different typologies (relational, verbal-emotional, and physical) and roles (non-perpetrator and non-victim, perpetrator, victim, and perpetrator-victim). Secondly, explore potential gender-based differences in sexism, empathy, and assertiveness and their potential associations with TDV perpetration and victimization. Lastly, develop separate models for each gender to examine the predictive role of sexism on TDV, with empathy and assertiveness as mediating variables.

## 2 Method

### 2.1 Participants

The study sample consisted of 1,650 students attending public secondary schools in various regions of Spain, namely Asturias, Murcia, Teruel, and Valencia. Out of the total sample, 862 students (49.80% males and 50.20% females) who reported having been in an intimate relationship within the past 12 months were included in the study. The mean age of the students was 14.13 years (range = 11–17, SD = 1.35), and they were enrolled in the first (15%), second (28.10%), third (28.10%), or fourth year (28.90%) of compulsory secondary education, which corresponds to grades 7, 8, 9, and 10 in the North American educational system.

### 2.2 Instruments

The Conflict in Adolescent Dating Relationships Inventory (CADRI, Wolfe et al., 2001), adapted to Spanish by Fernández-Fuertes et al. (2006), was used in this study. It comprises 25 items designed to assess conflictive actions within the past 12 months among adolescent dating partners who either engage in abusive behavior or are victimized. Respondents rated the frequency of experiencing these actions on a 4-point scale, ranging from 1 (never) to 4 (often). The inventory follows a five-factor structure representing five types of abuse: physical, sexual, threatening, relational, and emotional or verbal. Previous Spanish validation demonstrated that three factors showed optimal reliability: emotional violence (10 items, Cronbach’s alpha = 0.79), physical violence (4 items, Cronbach’s alpha = 0.76), and relational violence (3 items, Cronbach’s alpha = 0.73). In this study, all scales exhibited high reliability for both perpetration of TDV (Cronbach’s alpha = 0.83) and victimization of TDV (Cronbach’s alpha = 0.90).

The Adolescent Sexism Detection Scale (Recio et al., 2007) was utilized in the study. It comprises 26 items assessing Hostile Sexism (16 items) and Benevolent Sexism (10 items). Participants rated each item on a 6-point scale, ranging from 1 (strongly disagree) to 6 (strongly agree). Previous research conducted with Spanish adolescents reported good internal consistency with Cronbach’s alpha values ranging between 0.80 and 0.86 for Benevolent Sexism and between 0.92 and 0.94 for Hostile Sexism (Recio et al., 2007; Ramiro-Sánchez et al., 2018). In the present sample, Cronbach’s alpha coefficients were 0.93 for Hostile Sexism, 0.89 for Benevolent Sexism, and 0.89 for Trait Sexism.

The Interpersonal Reactivity Index (Davis, 1983), adapted to Spanish by Mestre et al. (2004), was administered in this study. It consists of 28 items that assess cognitive and emotional dimensions of empathy, including Perspective Taking (7 items), Fantasy (7 items), Empathic Concern (7 items), and Personal Distress (7 items). Respondents rated the extent to which each statement described themselves on a 5-point scale, ranging from 1 (does not describe me well) to 5 (describes me very well). Previous studies with Spanish adolescent samples reported Cronbach’s alpha values between 0.56 and 0.76 for the four subscales (Mestre et al., 2004). In our sample, the two reversed items of the Personal Distress subscale were removed due to reliability issues (Józsa and Morgan, 2017). The Cronbach’s alpha coefficients were 0.65 for Perspective Taking, 0.70 for Fantasy, 0.72 for Empathic Concern, and 0.72 for Personal Distress.

The Assertive Interpersonal Schema Questionnaire (Vagos and Pereira, 2010) was employed in this study to assess core beliefs about oneself, others, and social events or interactions. The questionnaire comprises 21 items, which measure four subscales: Outer Emotional Support (5 items), Practical Personal Ability (4 items), Interpersonal Management (8 items), and Affective Personal Ability (4 items). Participants were asked to rate the extent to which the statements described themselves on a 5-point scale, ranging from 1 (completely false to me) to 5 (completely true to me). Previous studies with Portuguese samples reported Cronbach’s alpha values between 0.75 and 0.83 for the subscales (Vagos and Pereira, 2010). In our sample, Cronbach’s alpha coefficients were 0.86 for Outer Emotional Support, 0.86 for Practical Personal Ability, 0.74 for Interpersonal Management, and 0.72 for Affective Personal Ability.

### 2.3 Procedure

To ensure a geographically diverse sample, authorization was obtained from the Directorates-General for Education of the Autonomous Communities of Comunidad Valenciana, Region of Murcia, Aragón, and Asturias. A total of 10 schools voluntarily participated in the study through convenience sampling. These schools represented both public ownership (82.73%; *n* = 1,365) and private-concerted ownership (17.27%; *n* = 285). Out of the total 1,650 participants, 45.63% (*n* = 753) attended schools in the province of Teruel (Aragón), spread across four schools; 32.9% (*n* = 543) attended schools in the Region of Murcia, also spread across four schools; 7.88% (*n* = 130) attended a school in Asturias; and 13.57% (*n* = 224) attended a school in the province of Valencia (Comunidad Valenciana).

A detailed letter summarizing the research project was sent to the selected schools before data collection. The researchers personally contacted the principals of these schools to provide a comprehensive explanation of the study’s purpose and to obtain permission to conduct the research. Informed consent was obtained from parents or guardians for their children’s participation in the study. The students themselves were fully informed about the study objectives and assured that participation was voluntary and anonymous. Data collection occurred during regular classroom sessions, specifically during a homeroom class, and completing the questionnaire took approximately 20 min. A researcher was present during the administration of the instruments to offer necessary support to the students. Participants were informed of their voluntary participation rights following the Spanish Organic Law 3/2018 on Personal Data Protection and Digital Rights Guarantee. The selection criteria for participation were as follows: (a) age between 11 and 17 years old; (b) explicit agreement to participate; and (c) proper completion of the survey.

The study adhered to The Code of Ethics of the World Medical Association (Declaration of Helsinki) and was approved by the Committee of Evaluation and Follow-up of Research with Human Beings (CEISH) from Valencian International University (protocol code CEID2020_03).

### 2.4 Data analysis

First, specific analyses were conducted to describe the results obtained by the male and female participants in the dependent variables of interest. Central tendency (mean) and dispersion (standard deviation) were calculated for continuous variables, while frequencies were determined for categorical variables. Group comparisons were performed using Student’s t-test, as the sample size allowed for parametric statistics. Effect sizes (Cohen’s *d*) were calculated to supplement the comparative analysis. Chi-square tests (*χ*^2^) were employed to compare categorical variables, such as gender and types of teen dating violence (TDV), with Cramer’s V selected as the effect size measure. The classification of participants into each TDV category was presented, along with the expected values for each case.

Bivariate correlations were examined using Pearson’s correlation coefficient (*r*), which served as the basis for mediation models. Model 4 of the SPSS Macro PROCESS (Hayes) was used for the mediation analysis, with sexism as the predictor variable and violence (perpetration or victimization) as the outcome variable. The factors from the empathy and assertiveness questionnaires were considered mediator variables after confirming their independent predictive capacity for other dimensions in the model. The statistical significance of these analyses was assessed using the bootstrapping method.

All statistical procedures were conducted using the Statistical Package for the Social Sciences (SPSS), version 25. A significance level of 0.05 was set for testing the null hypothesis.

## 3 Results

### 3.1 Descriptive and comparative analyses

A comparative analysis was conducted between males and females on the variables of TDV (Table 1), revealing statistically significant differences primarily in perpetration. Specifically, females obtained higher scores in verbal-emotional TDV perpetration (*t*_862_ = −4.60; *p* < 0.001) and the overall factor of TDV perpetration (*t*_862_ = −3.53; *p* < 0.001). Males scored higher in relational TDV perpetration (*t*_862_ = 3.04; *p* = 0.002). In all these cases, the effect size was small. Regarding TDV victimization, females obtained higher scores in verbal-emotional TDV (*t*_862_ = −2.86; *p* = 0.004).

**Table 1:** Comparison of TDV types (perpetrator and/or victim) based on gender.

	Males (*n* = 430)		Females (*n* = 434)			Sign/ES	
Factor	*M*	*SD*	*M*	*SD*	*t*	*p*-value	*d*
Perpetrated TDV	2.95	4.41	4.03	4.64	−3.53	0.000	0.239*
Relational TDV	0.33	0.82	0.19	0.54	3.04	0.002	0.202*
Verbal-emotional TDV	2.27	3.21	3.35	3.70	−4.60	0.000	0.312*
Physical TDV	0.35	1.02	0.50	1.19	−1.95	0.052	0.135
Received TDV	3.46	5.48	4.10	6.38	−1.60	0.110	0.108
Relational TDV	0.49	1.14	0.38	1.07	1.47	0.142	0.099
Verbal-emotional TDV	2.55	3.87	3.39	4.70	−2.86	0.004	0.195
Physical TDV	0.42	1.16	0.34	1.30	0.92	0.356	0.065

*M*, Mean; SD, Standard Deviation. Statistical significance (*t*-test): *p ≤ 0.05, **p ≤ 0.01, and ***p ≤ 0.001. Effect size: ES (Cohen’s *d*): *small, **medium, and ***large.

When comparing males and females in terms of TDV types (Table 2), significant differences were observed between the two groups (*χ*^2^ = 13.024, *p* = 0.005). Specifically, males scored higher than expected in the category of “neither perpetrator nor victim,” while females scored higher in “perpetrator and victim.” Despite the observed statistical significance, the effect size was negligible.

**Table 2:** Comparison of frequency distributions for TDV roles by gender.

	Male	Female	Chi^2^	*p*-value	*V*
Neither perpetrator nor victim	131 (112.5)	95 (113.5)	13.024	0.005	0.123
Perpetrator	41 (45.3)	50 (45.7)			
Victim	33 (26.9)	21 (27.1)			
Perpetrator and victim	225 (245.4)	268 (247.6)			

p, statistical significance; V, Cramer’s V.

Furthermore, when examining the relationship between gender and different modalities of sexism (Table 3), it was found that males had higher scores in hostile sexism (*t*_862_ = 6.16; *p* < 0.001) and benevolent sexism (*t*_862_ = 2.60; *p* = 0.009). The effect sizes were small for hostile sexism (*d* = 0.420) and negligible for benevolent sexism.

**Table 3:** Comparison of sexism by gender.

	Males		Females			Sign/ES	
Factor	*M*	*SD*	*M*	*SD*	*t*	*p*-value	*d*
Hostile sexism	31.17	14.70	25.46	12.42	6.16	0.000	0.420*
Benevolent sexism	26.46	10.81	24.53	11.05	2.60	0.009	0.177

M, Mean; SD, Standard deviation. Statistical significance (*t*-test): *p ≤ 0.05, **p ≤ 0.01, and ***p ≤ 0.001. Effect size: ES (Cohen’s *d*): *small, **medium, and ***large.

Regarding empathy (Table 4), it was observed that in all cases, females obtained higher scores than males: perspective taking (*t*_862_ = −2.83; p = 0.005), fantasy (*t*_862_ = −5.68; *p* < 0.001), empathic concern (t_862_ = −3.64; *p* < 0.001), and personal distress (*t*_862_ = −3.55; *p* < 0.001). The effect sizes were small for fantasy (*d* = 0.385), empathic concern (*d* = 0.246), and personal distress (*d* = 0.242). The effect size for perspective-taking was negligible (*d* = 0.194).

**Table 4:** Comparison of empathy by gender.

	Males		Females			Sign/ES	
Factor	*M*	*SD*	*M*	*SD*	*t*	*p*-value	*d*
Perspective taking	19.58	5.11	20.54	4.80	−2.83	0.005	0.194
Fantasy	16.74	5.64	18.87	5.41	−5.68	0.000	0.385*
Empathic concern	19.82	5.44	21.09	4.87	−3.64	0.000	0.246*
Personal distress	16.35	5.08	17.51	4.50	−3.55	0.000	0.242*

M, Mean; SD, Standard Deviation. Statistical significance (*t*-test): *p ≤ 0.05, **p ≤ 0.01, and ***p ≤ 0.001. Effect size: ES (Cohen’s *d*): *small, **medium, and ***large.

Finally, assertiveness dimensions were compared between both genders (Table 5), and it was found that females obtained higher scores in external emotional support (*t*_862_ = −4.76; *p* < 0.001), interpersonal management (*t*_862_ = −2.06; *p* = 0.040), and affective personal ability (*t*_862_ = −2.15; *p* = 0.032). Only external emotional support showed an appropriate effect size but of a small magnitude (*d* = 0.324).

**Table 5:** Comparison of assertiveness by gender.

	Males		Females			Sign/ES	
Factor	*M*	*SD*	*M*	*SD*	*t*	*p*-value	*d*
External emotional support	21.02	4.62	22.38	3.74	−4.76	0.000	0.324*
Practical personal ability	16.46	3.72	16.37	3.76	0.34	0.732	0.024
Interpersonal management	30.75	6.00	31.52	4.99	−2.06	0.040	0.140
Affective personal ability	16.42	3.43	16.90	3.05	−2.15	0.032	0.148

M, Mean; SD, Standard Deviation. Statistical significance (*t*-test): *p ≤ 0.05, **p ≤ 0.01, and ***p ≤ 0.001. Effect size: ES (Cohen’s *d*): *small, **medium, and ***large.

### 3.2 Correlational analyses

Separate analyses were conducted for males and females. In the male group (Table 6), it was observed that both perpetration and victimization of TDV positively correlated with all forms of sexism, assertiveness, and certain empathy factors (fantasy and personal distress). Significant negative correlations were found between all dimensions of sexism and assertiveness. Regarding empathy, negative associations were specifically observed between hostile sexism and the empathic concern subscale. Additionally, empathy showed positive correlations with all dimensions of assertiveness, except for personal distress, which did not exhibit any association with personal abilities (practical and affective).

**Table 6:** Correlation analysis (male sample).

	TDVP	TDVV	TSEX	HSEX	BSEX	EMPT	EMFA	EMEC	EMPD	ASES	ASPA	ASIM	ASAPA
TDVP	/												
TDVV	0.710***	/											
TSEX	0.252***	0.186***	/										
HSEX	0.256***	0.180***	0.951***	/									
BSEX	0.206***	0.164***	0.908***	0.735***	/								
EMPT	0.026	0.078	−0.012	−0.026	0.009	/							
EMFA	0.113*	0.115*	0.016	−0.011	0.049	0.612***	/						
EMEC	0.031	0.090	−0.077	−0.113*	−0.016	0.709***	0.688***	/					
EMPD	0.153***	0.161***	0.178***	0.162***	0.170***	0.641***	0.626***	0.640***	/				
ASES	−0.181***	−0.103*	−0.227***	−0.236***	−0.179***	0.307***	0.171***	0.277***	0.125**	/			
ASPA	−0.214***	−0.141**	−0.240***	−0.229***	−0.217***	0.141**	0.117*	0.181***	−0.012	0.583***	/		
ASIM	−0.212***	−0.151**	−0.231***	−0.237***	−0.186***	0.325***	0.245***	0.300***	0.157***	0.743***	0.700***	/	
ASAPA	−0.171***	−0.070	−0.205***	−0.217***	−0.156***	0.211***	0.147**	0.218***	0.083	0.735***	0.720***	0.755***	/

TDVP, Teen dating violence (perpetration); TDVV, Teen dating violence (victimization); TSEX, Total sexism; HSEX, Hostile sexism; BSEX, Benevolent sexism; EMPT, Empathy (perspective taking); EMFA, Empathy (fantasy); EMEC, Empathy (empathic concern); EMPD, Empathy (personal distress); ASES, Assertiveness (external emotional support); ASPA, Assertiveness (practical personal ability); ASIM, Assertiveness (interpersonal management); ASAPA, Assertiveness (affective personal ability). Statistical significance (Student’s *t*-test): *p ≤ 0.05, **p ≤ 0.01, and ***p ≤ 0.001.

In the female group (Table 7), positive associations were observed between TDV (perpetration and victimization) and all types of sexism, along with negative correlations between TDV dimensions and assertiveness. Sexism was directly related to personal distress and inversely related to assertiveness, except for benevolent sexism, which showed no association with interpersonal management. Empathy, perspective-taking, and empathic concern were positively associated with different forms of assertiveness (external emotional support, practical personal ability, and interpersonal management).

**Table 7:** Correlation analysis (female sample).

	TDVP	TDVV	TSEX	HSEX	BSEX	EMPT	EMFA	EMEC	EMPD	ASES	ASPA	ASIM	ASAPA
TDVP	/												
TDVV	0.658***	/											
TSEX	0.172***	0.146**	/										
HSEX	0.142***	0.113*	0.946***	/									
BSEX	0.184***	0.165***	0.931***	0.762***	/								
EMPT	−0.005	0.051	0.022	0.012	0.031	/							
EMFA	0.019	0.020	−0.040	−0.069	−0.003	0.447***	/						
EMEC	−0.043	−0.019	0.036	0.030	0.039	0.615***	0.475***	/					
EMPD	0.079	0.095*	0.162***	0.140**	0.165***	0.474***	0.495***	0.577***	/				
ASES	−0.153***	−0.160***	−0.204***	−0.181***	−0.204***	0.110*	−0.067	0.141**	−0.085	/			
ASPA	−0.198***	−0.163***	−0.124**	−0.133**	−0.098*	0.107*	−0.006	0.131**	−0.066	0.417***	/		
ASIM	−0.097*	−0.134**	−0.106*	−0.118*	−0.079	0.118*	0.000	0.138**	−0.012	0.548***	0.589***	/	
ASAPA	−0.105*	−0.148**	−0.119*	−0.120*	−0.103*	0.068	−0.059	0.068	−0.116*	0.588***	0.571***	0.660***	/

TDVP, Teen dating violence (perpetration); TDVV, Teen dating violence (victimization); TSEX, Total sexism; HSEX, Hostile sexism; BSEX, Benevolent sexism; EMPT, Empathy (perspective taking); EMFA, Empathy (fantasy); EMEC, Empathy (empathic concern); EMPD, Empathy (personal distress); ASES, Assertiveness (external emotional support); ASPA, Assertiveness (practical personal ability); ASIM, Assertiveness (interpersonal management); ASAPA, Assertiveness (affective personal ability). Statistical significance (Student’s *t*-test): *p ≤ 0.05, **p ≤ 0.01, and ***p ≤ 0.001.

### 3.3 Mediational analyses

**Figure 1:**
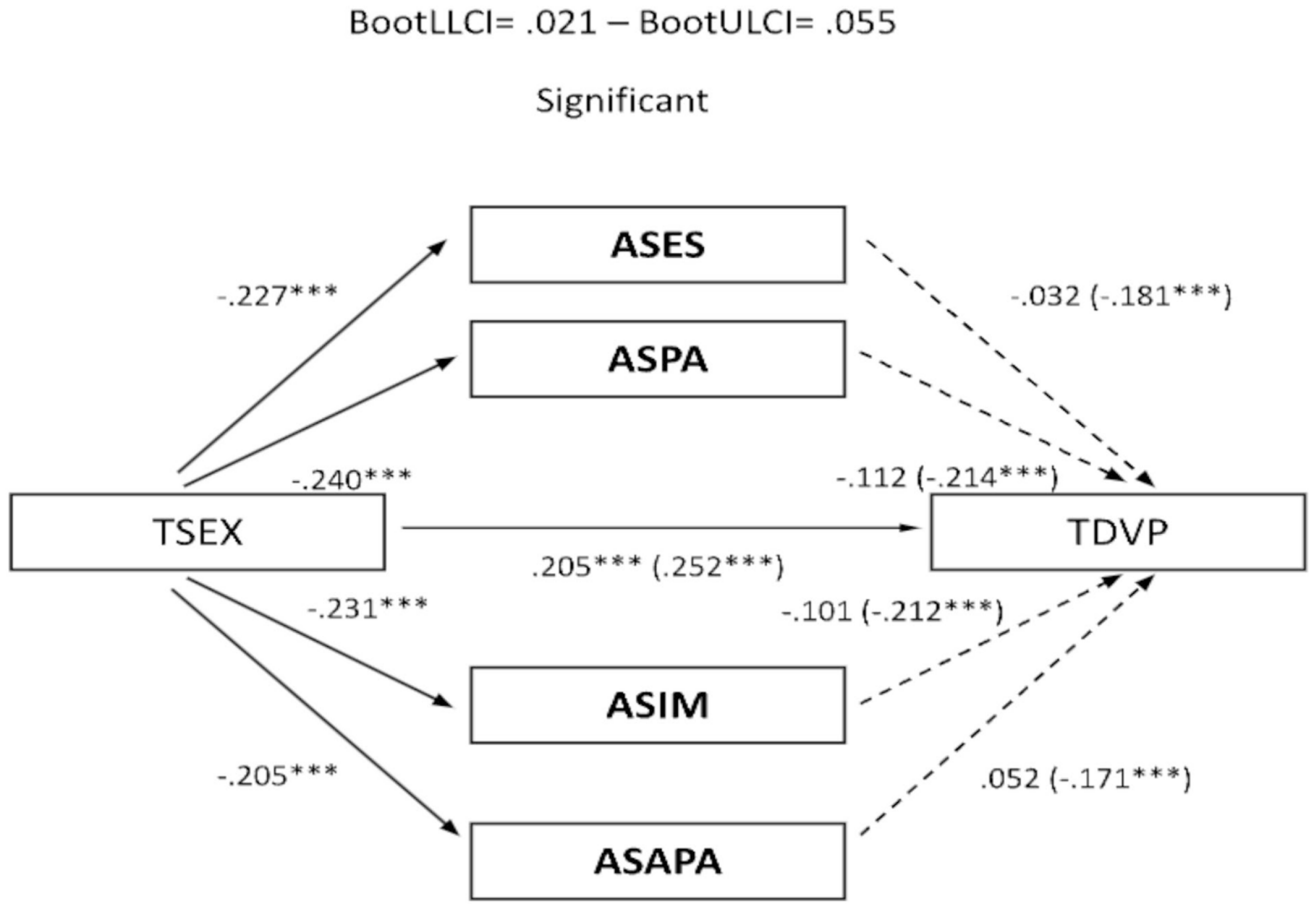
Mediation analysis (Teen dating violence – perpetration as the outcome variable and assertiveness as the predictor variable): males: TSEX, Total Sexism; ASES, Assertiveness (external emotional support); ASPA, Assertiveness (practical personal ability); ASIM, Assertiveness (interpersonal management); ASAPA, Assertiveness (affective personal ability); TDVP, Teen Dating Violence (perpetration). Statistical significance (Student’s *t*-test): *p ≤ 0.05, **p ≤ 0.01, and ***p ≤ 0.001.

**Figure 2:**
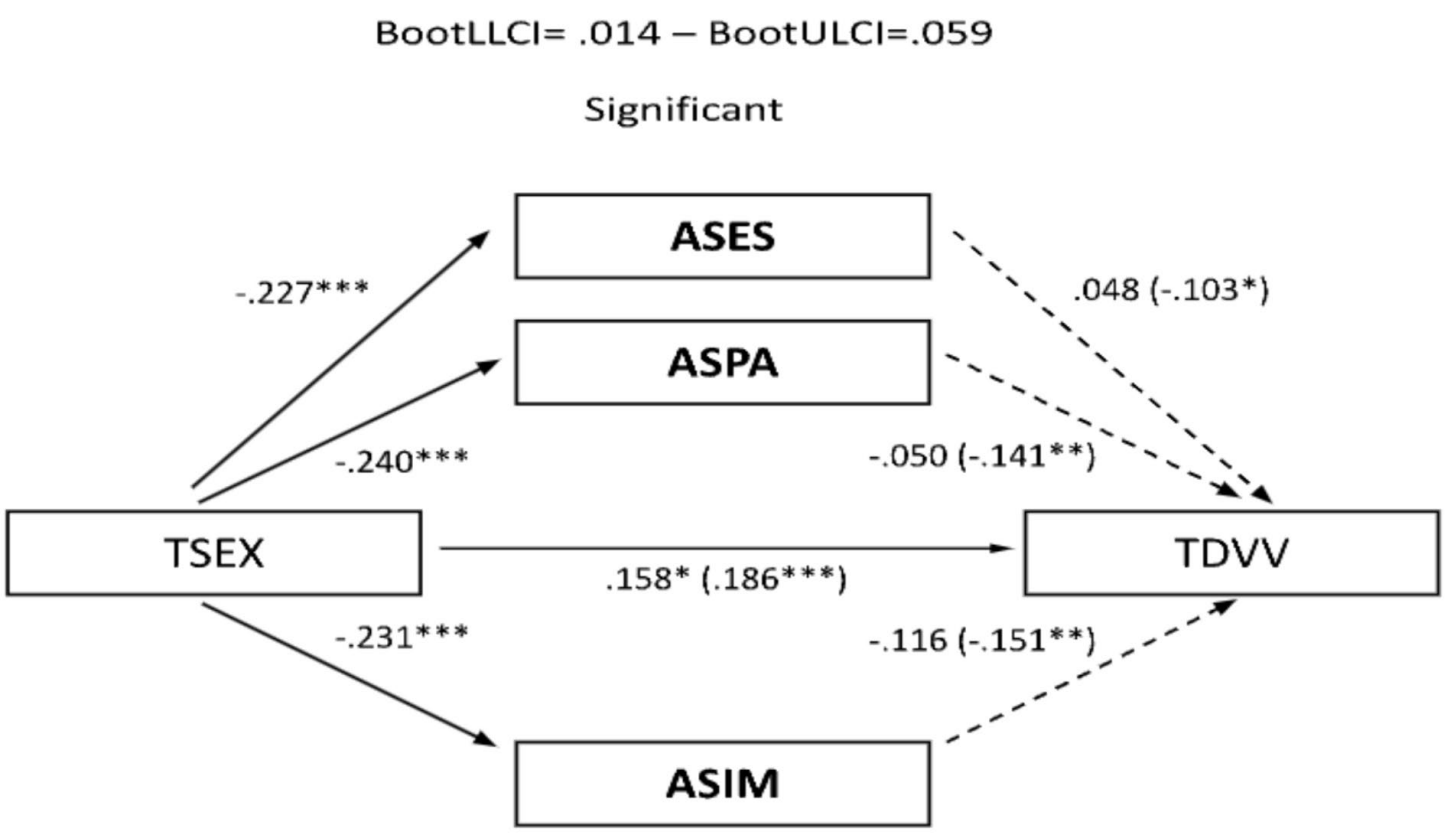
Mediation analysis (teen dating violence – victimization as the outcome variable and assertiveness as the predictor variable): males: TSEX, Total Sexism; ASES, Assertiveness (external emotional support); ASPA, Assertiveness (practical personal ability); ASIM, Assertiveness (interpersonal management); TDVV, Teen Dating Violence (victimization). Statistical significance (Student’s *t*-test): *p ≤ 0.05, **p ≤ 0.01, and ***p ≤ 0.001.

**Figure 3:**
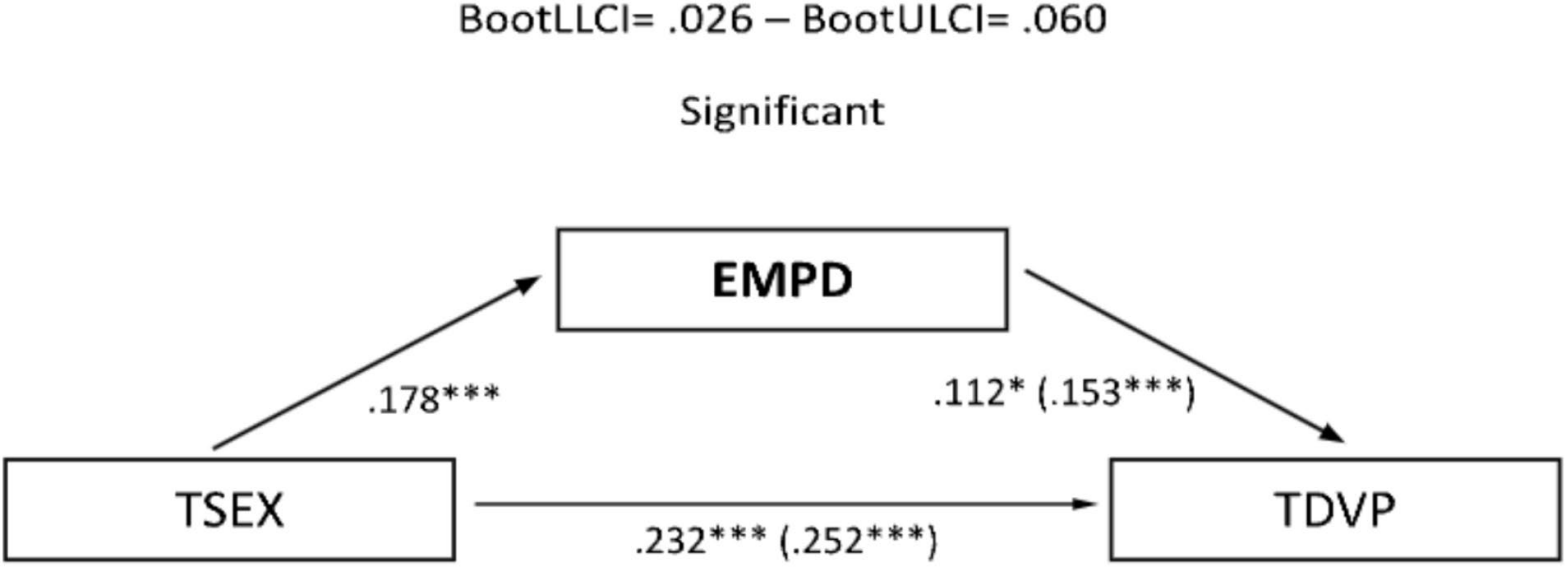
Mediation analysis (teen dating violence – perpetration as the outcome variable and empathy as the predictor variable): males: TSEX, Total Sexism; EMPD, Empathy (personal distress); TDVP, Teen Dating Violence (perpetration). Statistical significance (Student’s *t*-test): *p ≤ 0.05, **p ≤ 0.01, and ***p ≤ 0.001.

**Figure 4:**
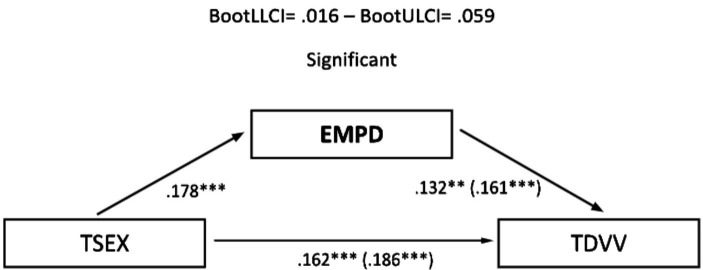
Mediation analysis (teen dating violence – victimization as the outcome variable and empathy as the predictor variable): males: TSEX, Total Sexism; EMPD, Empathy (personal distress); TDVV, Teen Dating Violence (victimization). Statistical significance (Student’s *t*-test): *p ≤ 0.05, **p ≤ 0.01, and ***p ≤ 0.001.

The significant correlations described allowed the development of mediational models, with sexism as the predictor variable and TDV perpetration and victimization as outcome variables. Assertiveness and empathy served as mediating variables. In the male sample, no relevant mediation effects were observed for assertiveness (Figures 1, 2). Nevertheless, a partial mediation effect was established through personal distress (Figures 3, 4), indicating a statistically significant mediating role between sexism and TDV, encompassing both perpetration and victimization.

**Figure 5:**
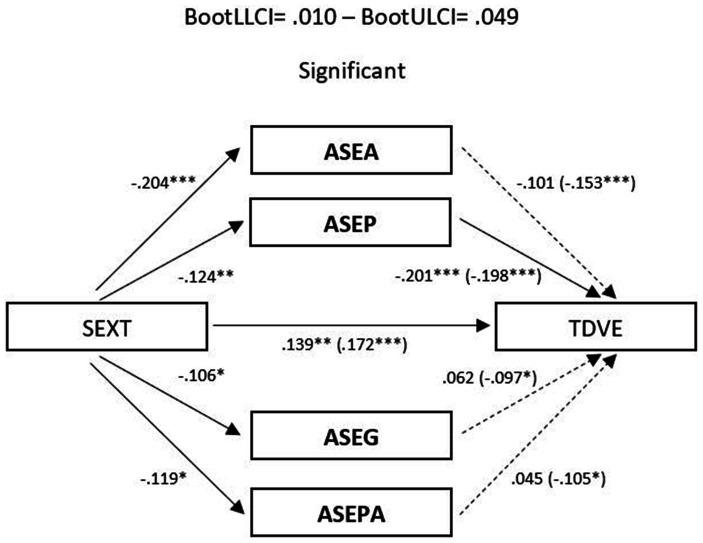
Mediation analysis (teen dating violence – perpetration as the outcome variable and assertiveness as the predictor variable): females: TSEX, Total Sexism; ASES, Assertiveness (external emotional support); ASPA, Assertiveness (practical personal ability); ASIM, Assertiveness (interpersonal management); ASAPA, Assertiveness (affective personal ability); TDVP, Teen dating violence (perpetration). Statistical significance (Student’s *t*-test): *p ≤ 0.05, **p ≤ 0.01, and ***p ≤ 0.001.

**Figure 6:**
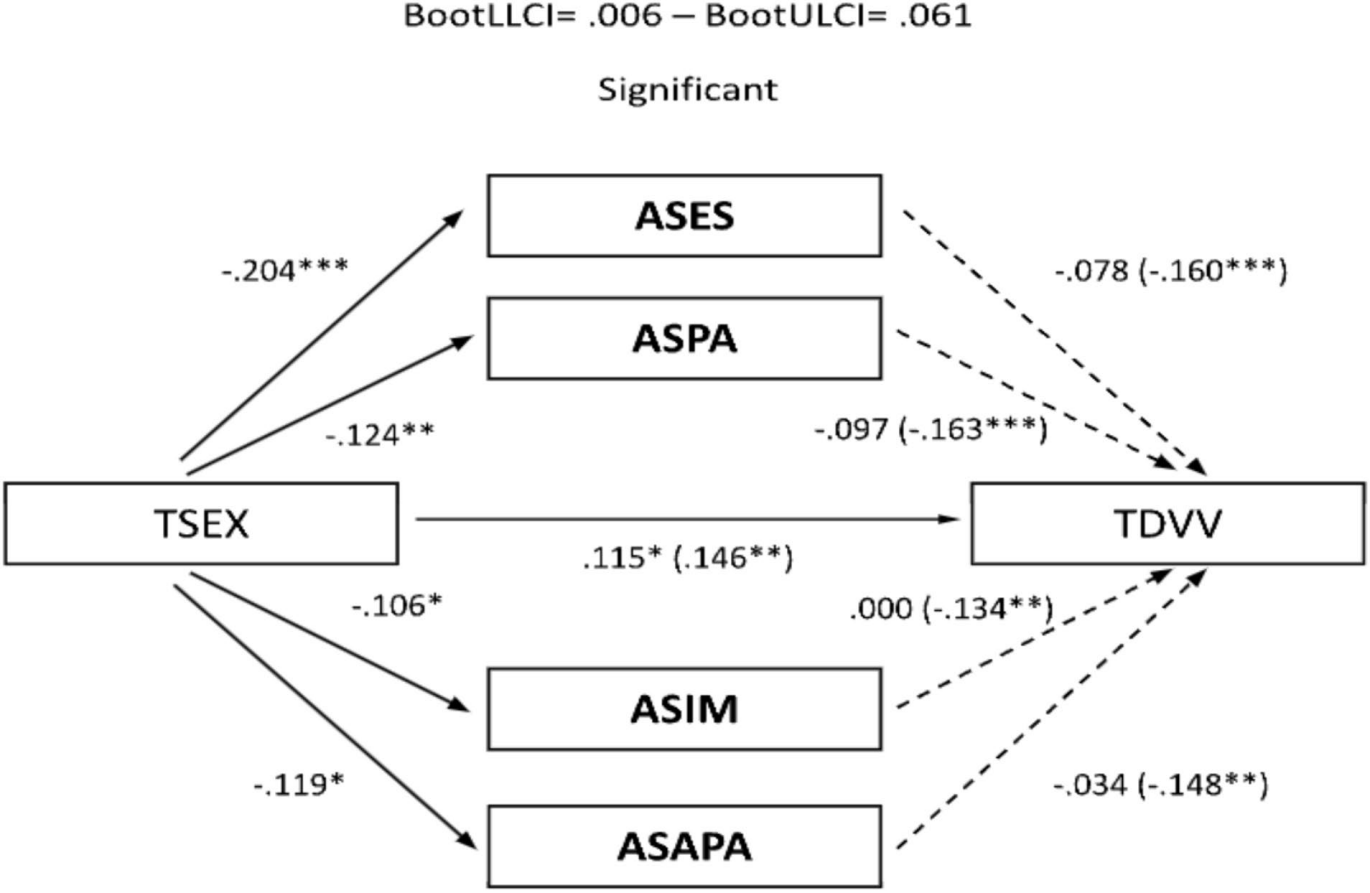
Mediation analysis (teen dating violence – victimization as the outcome variable and assertiveness as the predictor variable): females: TSEX, Total Sexism; ASES, Assertiveness (external emotional support); ASPA, Assertiveness (practical personal ability); ASIM, Assertiveness (interpersonal management); ASAPA, Assertiveness (affective personal ability); TDVV, Teen dating violence (victimization). Statistical significance (Student’s *t*-test): *p ≤ 0.05, **p ≤ 0.01, and ***p ≤ 0.001.

**Figure 7:**
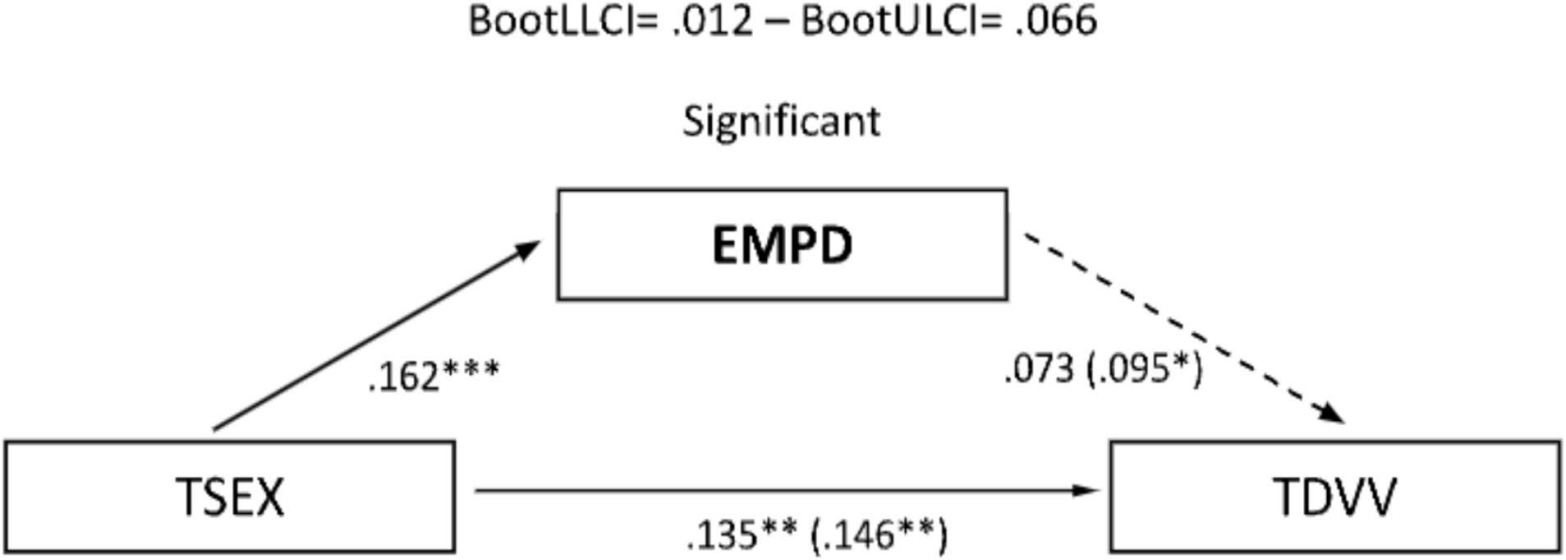
Mediation analysis (teen dating violence – victimization as the outcome variable and empathy as the predictor variable): females: TSEX, Total Sexism; EMPD, Empathy (personal distress); TDVV, Teen dating violence (victimization). Statistical significance (Student’s *t*-test): *p ≤ 0.05, **p ≤ 0.01, and ***p ≤ 0.001.

In the sample of women, a partial mediation effect for assertiveness (practical personal ability) was observed on the established relationship between sexism and TDV perpetration (Figure 5), although this was not replicated in the same way when TDV (victimization) was used as the outcome variable (Figure 6). Regarding the mediating role of personal distress, it could only be applied in the case of the female sample on TDV victimization, as the variables involved did not present a relevant association with TDV perpetration. In any case, empathy did not generate a relevant mediation effect in this case (Figure 7).

## 4 Discussion and findings

Taking into account early experiences in romantic relationships of adolescents that can establish a pattern rooted in violence and unhealthy relationships (Muñoz-Rivas et al., 2015), the present study has proposed a predictive models of sexism differentiated for perpetration and victimization of TDV, with empathy and assertiveness as mediating variables for both genders.

The first intermediate objective of this study is to examine gender differences in the perpetration and victimization of TDV, including its typologies (relational, verbal-emotional, and physical) and different roles (non-perpetrator/non-victim, perpetrator, victim, and perpetrator-victim). Previous research has reported a higher prevalence of perpetrated TDV among females (Valdivia-Salas et al., 2021), while other studies suggest that males are more likely to engage in violent behaviors within romantic relationships (Dosil et al., 2020). Consistent with these findings, females exhibit higher levels of verbal-emotional TDV, whereas males are more likely to engage in relational violence (Courtain and Glowacz, 2021; Valdivia-Salas et al., 2021). Regarding victimization, no significant gender differences are observed in total scores or types of victimization, aligning with the bidirectionality of TDV found in previous studies (Wincentak et al., 2017). However, conflicting findings have also been reported, with some studies indicating higher levels of victimization among females (Vives-Cases et al., 2021), and others suggesting higher victimization rates among males (Dosil et al., 2022).

In relation to the roles of perpetrator/victim, females show a higher prevalence of the combined role, although the effect size is negligible. These findings are supported by previous studies (Ruiz et al., 2010; Ybarra et al., 2016; Rubio-Garay et al., 2017; Wincentak et al., 2017; Karsberg et al., 2018). Bidirectional violence may be related to limited previous experience in conflict resolution within dating relationships (Viejo et al., 2016). However, it is important to note that the severity of such aggressions may impact females more due to the prevailing gender inequality in society (Cross and Overall, 2018; Dosil et al., 2022).

Considering this background, it is crucial for studies on TDV to examine the relationship between socio-emotional skills and sexist attitudes from a gender perspective (Ayala et al., 2021; Madrona-Bonastre et al., 2023). Accordingly, the second intermediate objective of this research was to analyze significant differences in sexism, empathy, and assertiveness based on gender. For sexism, the data indicate that males tend to have higher scores in hostile sexism than females (Brandt, 2011; Barreto and Doyle, 2023). Conversely, no significant differences are observed in benevolent sexism (Barreto and Doyle, 2023). This could be because hostile sexism behaviors directly exhibit hatred and rejection toward females (Glick and Fiske, 2011), making them more visibly disapproved by society and potentially inhibiting the expression of benevolent sexism. Benevolent sexism, being more subtle and paternalistic, may go unnoticed (Glick and Fiske, 2011). Thus, the absence of differences in benevolent sexism between males and females found in this study may be rooted in adolescents’ social acceptance of such behaviors. On the other hand, the small-sized differences in hostile sexism between males and females may be related to sexist behaviors where aggressiveness is more evident in males, facilitating its expression. However, the current evidence is inconsistent, as some studies indicate that males score higher in benevolent sexism than females (Ayala et al., 2021). Therefore, further studies are necessary to better understand the reasons behind accepting or rejecting sexist beliefs based on gender.

Regarding empathy, the data show that females have higher levels of fantasy, empathic concern, and personal distress than males. Previous studies suggest that females exhibit higher affective and cognitive empathy (Cebollero-Salinas et al., 2022). This fact could be related to gender socialization, which promotes greater emotional skills in females (Overgaauw et al., 2017). Similarly, in the meta-analysis by Abramson et al. (2020) on the genetics of empathy research, it is concluded that regardless of sex and age, only cognitive empathy appears susceptible to shared environmental factors.

When considering gender differences in assertiveness, it is observed that females tend to exhibit higher levels of external emotional support compared to males. This difference may be attributed to coping strategies influenced by gender socialization, with females displaying a greater inclination to seek emotional support (Camara et al., 2017). Furthermore, the significance of peer support in understanding females’ involvement in both victimization and perpetration of TDV has been demonstrated by Richards and Branch (2012). In contrast, the role of social support from friends and family in influencing males’ engagement in partner violence may be relatively smaller. Importantly, Vagos and Pereira (2022) found no significant gender differences in assertiveness.

Finally, concerning the main objective, the predictive models of sexism differentiated for perpetration and victimization of TDV, with empathy and assertiveness as mediating variables for both genders, revealed that sexism, assertiveness, and empathy individually exerted statistically significant prediction effects on TDV. Furthermore, the mediation effects of these dimensions in the relationship between sexism and TDV displayed a differentiated pattern based on gender. Specifically, personal distress partially mediated the relationship between sexism and the perpetration and victimization of TDV in males, highlighting it as a key therapeutic target for potential preventive interventions. However, this effect was not observed in females, where partial mediation was found in the practical personal ability dimension for the relationship between sexism and the perpetration of TDV. These findings underscore the importance of emphasizing emotional information processing in males and communication patterns in females to guide the development of intervention proposals.

From this perspective, the assimilation of sexist attitudes and beliefs in society plays a crucial role in shaping the dynamics of adolescent romantic relationships (Arenas, 2013). According to Fernández et al. (2020), the acceptance of violence and sexism alone can be considered risk factors in young romantic relationships. However, these variables further define the risk when combined with the victim role. Consistent with previous research (Arnoso et al., 2017; Cuadrado-Gordillo and Martín-Mora-Parra, 2022), the findings support the notion that sexism, although not a causal element, increases the likelihood of perpetrating violence in relationships when interacting with other factors. Several studies provide support for the association between victimization in relationships and sexist beliefs, which serve as facilitating factors for the initiation and perpetuation of TDV (Fernández-Fuertes et al., 2018; Marcos et al., 2020; Rey-Anacona and Martínez-Gómez, 2022). Valdivia-Salas et al. (2021) demonstrated a positive relationship between high personal distress and TDV. Furthermore, evidence suggests that perpetrators and victims of TDV exhibit high levels of benevolent sexism and low levels of emotional regulation skills and practical personal ability (Valdivia-Salas et al., 2023). In line with the present study, Xia et al. (2018) proposed that assertiveness may be a valuable interpersonal skill in promoting healthy romantic relationships. Consequently, initiatives aimed at preventing teen dating violence should not only focus on reducing violent behaviors but also on fostering the development of positive relational skills within adolescent dating relationships.

About the limitations of this study, it is important to note that its design is cross-sectional, which precludes the establishment of causal relationships. Future research should consider employing longitudinal methodologies to determine the directionality of the observed effects. A convenience sample may also introduce sampling bias, although efforts were made to include participants from diverse geographic areas. Moreover, the reliance solely on self-report questionnaires may introduce social desirability biases. It would be valuable to incorporate other assessment tools and gather perspectives from families and teachers to enhance the validity of the findings. Another limitation pertains to the variables related to adolescent romantic relationships, as this study did not consider sexual orientation or relationship duration. These aspects should be considered in future investigations, the differentiation between homosexual and heterosexual population should be taken into account. Regarding the duration of the relationship, future research could employ mediation models that account for the frequency and severity of TDV and the different roles (perpetrator, victim, perpetrator-victim) assumed by individuals within their romantic relationships. Notably, previous studies, including the present research, have observed that the prevalent role in this type of relationship is that of an occasional perpetrator-victim, with similar prevalence among males and females. This role is associated with low interpersonal skills due to limited experience in conflict resolution. Therefore, examining variables such as assertiveness and empathy is important to understand their impact on TDV across different roles. Furthermore, including an analysis of sexist beliefs concerning TDV is crucial, as these beliefs may contribute to minimizing or concealing such violence and could serve as risk factors for developing future violent behaviors in adulthood. It is worth highlighting the importance of online relationships among adolescents today, and it would be very interesting to follow this line of research in future studies.

In conclusion, the present study addresses the three research questions posed, which collectively proposed an explanatory model of TDV, considering sexism as a predictor variable, and empathy and assertiveness as mediating variables. It also raises the possibility that the differences found in previous literature regarding gender differences for all these variables might suggest the convenience of proposing differentiated models based on gender. This study provides valuable insights into TDV by emphasizing the importance of addressing sexist beliefs among adolescents, regardless of gender, with specific attention to males. Sexism emerges as a robust predictor of both TDV perpetration and victimization, contributing to the justification of these behaviors. A comprehensive affective-relational education program for adolescents should promote diverse social skills, including empathy and assertiveness, which have been identified as crucial prosocial skills in psychoeducational and preventive interventions. Furthermore, this study supports previous findings indicating that certain dimensions of these skills mediate the relationship between sexism and TDV perpetration or victimization. Specifically, personal distress mediates the effects in male perpetrators and victims, while practical personal ability mediates the relationship in female perpetrators. Therefore, future preventive measures should carefully consider these dimensions, moving beyond the simplistic view of empathy and assertiveness as unidimensional skills. By addressing sexist beliefs, fostering diverse social skills, and targeting underlying factors associated with TDV, effective prevention strategies can be developed to promote healthy and respectful relationships among adolescents.

## Data availability statement

The original contributions presented in the study are included in the article/supplementary material, further inquiries can be directed to the corresponding authors.

## Ethics statement

The study adhered to The Code of Ethics of the World Medical Association (Declaration of Helsinki) and was approved by the Committee of Evaluation and Followup of Research with Human Beings (CEISH) from Valencian International University (protocol code CEID2020_03). The studies were conducted in accordance with the local legislation and institutional requirements. Informed consent for participation was obtained from the participants’ legal guardians/next of kin.

## Author contributions

VJV-B: Conceptualization, Project administration, Resources, Supervision, Validation, Visualization, Writing – original draft, Writing – review & editing, Data curation, Formal analysis, Investigation, Methodology, Software. BI: Conceptualization, Visualization, Writing – original draft, Writing – review & editing. JM-M: Visualization, Writing – original draft, Writing – review & editing, Formal analysis. LC: Visualization, Writing – original draft, Writing – review & editing, Conceptualization. SG-M: Visualization, Writing – original draft, Writing – review & editing. MC-M: Visualization, Writing – original draft, Writing – review & editing. MªM: Visualization, Writing – original draft, Writing – review & editing. MªH-J: Visualization, Writing – original draft, Writing – review & editing, Conceptualization, Funding acquisition, Project administration, Resources, Supervision, Validation.
